# Application of Direct Sonoporation from a Defined Surface Area of the Peritoneum: Evaluation of Transfection Characteristics in Mice

**DOI:** 10.3390/pharmaceutics11050244

**Published:** 2019-05-22

**Authors:** Koyo Nishimura, Keita Yonezawa, Shintaro Fumoto, Yusuke Miura, Masayori Hagimori, Koyo Nishida, Shigeru Kawakami

**Affiliations:** 1 Department of Pharmaceutical Informatics, Graduate School of Biomedical Sciences, Nagasaki University, 1-7-1 Sakamoto, Nagasaki-shi, Nagasaki 852-8588, Japan; bb55316404@ms.nagasaki-u.ac.jp (K.N.); k.yonezawa0226@gmail.com (K.Y.); usk.mir@gmail.com (Y.M.); hagimori@nagasaki-u.ac.jp (M.H.); 2 Department of Pharmaceutics, Graduate School of Biomedical Sciences, Nagasaki University, 1-7-1 Sakamoto, Nagasaki-shi, Nagasaki 852-8588, Japan; sfumoto@nagasaki-u.ac.jp (S.F.), koyo-n@nagasaki-u.ac.jp (K.N.)

**Keywords:** nanobubbles, ultrasound, sonoporation, gene transfection, peritoneal mesothelial cells

## Abstract

In the present study, we developed a sonoporation system, namely “direct sonoporation”, for transfecting the peritoneum from a defined surface area to avoid systematic side effects. Here, the transfection characteristics are explained because there is less information about direct sonoporation. Naked pDNA and nanobubbles were administered to diffusion cell attached to the visceral and parietal peritoneum from the liver and peritoneal wall surface, respectively. Then, ultrasound was irradiated. Direct sonoporation showed a higher transfection efficacy at the applied peritoneum site from the liver surface while other sites were not detected. Moreover, transgene expression was observed in the peritoneal mesothelial cells (PMCs) at the applied peritoneum site. No abnormality was observed in the inner part of the liver. Although transgene expression of the visceral peritoneum was tenfold higher than that of the parietal peritoneum, transgene expression was observed in the PMCs on both the applied peritoneum sites. These results suggest that direct sonoporation is a site-specific transfection method of the PMCs on the applied peritoneum site without transgene expression at other sites and show little toxicity in the inner tissues at the applied site via cavitation energy. This information is valuable for the development of an intraperitoneal sonoporation device for treatment of peritoneal diseases such as peritoneal fibrosis.

## 1. Introduction

The peritoneum is composed of peritoneal mesothelial cells (PMCs) and a submesothelial layer as stroma. The peritoneum is a membrane that covers peritoneal tissues (liver, stomach etc.) and plays important roles such as protecting the intraperitoneal tissues, transporting low molecular compounds, and regulating peritoneal inflammation [1]. Moreover, the peritoneum is used as a peritoneal dialysis membrane for treatment of patients with end-stage renal failure. Long-term peritoneal dialysis causes peritoneal inflammation and/or peritoneal fibrosis [2,3,4]. So far, effective therapeutic method for peritoneal fibrosis have not been developed, and it is difficult to continue with peritoneal dialysis. Therefore, it is necessary to develop a therapeutic method for treatment of peritoneal fibrosis.

Intraperitoneal gene delivery might be an effective therapeutic method for treatment of peritoneal fibrosis. Several studies have developed an intraperitoneal injection-based gene delivery system using a virus vector or a non-virus vector such as adenovirus, cationic liposomes, etc. [5,6,7,8,9]. With respect to the non-virus transfection method, sonoporation can enable transfection into the ultrasound (US) irradiated site such as brain [10], liver [11], kidney [12] etc. because the cavitation energy creates transient pores on the cellular membrane [13,14]. An intraperitoneal sonoporation system that uses nano/microbubbles to achieve a high transfection efficacy to entire peritoneal tissues has been demonstrated [15,16]. Moreover, we revealed that the transgene expression after sonoporation may be in the PMCs after intraperitoneal administration. However, plasmid DNA (pDNA) can be distributed to an extensive area in the peritoneal cavity. Therefore, the site of transgene expression cannot be regulated. Whereby, even if the unexpected side-effects are accrued such as cancer, it is difficult to specify/remove transfection site. Therefore, for treatment of peritoneal fibrosis, a therapeutic gene transfected from a defined area is desired. The use of an intraperitoneal injection may have applications in the development of a medical device such as a peritoneal endoscope with a US irradiation probe. Because the technique that direct laparoscopic US irradiation to intraperitoneal tissues has been developed [17,18], the application of direct sonoporation with a medical device could be implemented. On the other hand, fibrosis may make US transmission difficult due to the thickening of the stroma. Thus, it is desire that transfection of pDNA consisting the secretory therapeutic protein such as hepatocyte growth factor (HGF) and bone marrow protein-7 (BMP-7) [19,20] gene to the appropriate site by sonoporation, and then therapeutic protein were expressed to the entire peritoneal cavity from the applied site.

Considering use of the medical device in the future, we have developed an in-situ gene transfection method by tissue surface administration of naked pDNA via the peritoneal tissues using a diffusion cell [21]. Tissue surface administration can transfer the gene to the defined area via the attached diffusion cell. Thus, we proposed that “direct sonoporation”, transfecting to the PMCs on the defined peritoneal surface area by the combination of sonoporation and tissue surface administration. As bubbles, microbubbles may be unstable compared with nanobubbles [22] and cationic bubbles may induce the cytotoxicity due to their cationic charge [11,23]. In addition, cationic bubbles/pDNA complex is considered that remaining complex could be transferred to the blood circulation and transfected in the undesired site. Because these reasons, we chose neutral nanobubbles for direct sonoporation. Direct sonoporation is expected to have few systematic side effects as it avoids transfer to the blood vesicle from the peritoneal cavity because transgene expression is in only applied site. However, the distribution of transgene expression and toxicity at the inner part of the peritoneal tissue may be occurred because cavitation energy of sonoporation can promote altering membrane permeability and/or opening of cell-cell junction [24,25,26]. Moreover, the transfection characteristics could be altered based on the applied tissue. Therefore, it is necessary to study the transgene expression characteristics not only in the systemic tissues but also its spatial distribution in the peritoneum after application of direct sonoporation for the development of a transfection system using a medical device in the peritoneum. Moreover, the transgene expression and toxicity inside the peritoneal tissues also needs to be investigated. However, there is little information available regarding direct sonoporation to the peritoneum surface area. 

The purpose of the present study is to clarify the transfection characteristics such as transgene expression and toxicity after direct sonoporation to a defined area in the peritoneum of mice. The spatial transgene expression characteristics and the injury to inner part of the liver can be easily evaluated. Therefore, the visceral peritoneum on the liver surface was selected as the site for application of direct sonoporation to investigate the systematic distribution, the depth of transgene expression, and hepatic toxicity after its application. Moreover, the transgene expression characteristics were also evaluated at different sites of the peritoneum to compare its results with those obtained using the parietal peritoneum.

## 2. Materials and Methods

### 2.1. pDNA

The firefly luciferase gene expression vector driven by a cytomegalovirus (CMV) promoter, pCMV-luciferase, was constructed as previously mentioned [27]. The pZsGreen1-N1 encoding the green fluorescent protein ZsGreen1 was purchased from Takara Bio Inc. (Shiga, Japan). The pCMV-luciferase and pZsGreen1-N1 were amplified in *Escherichia coli* strain DH5α. After isolation, pDNA was purified using an Endofree Plasmid Giga Kit (QIAGEN GmbH, Hilden, Germany). The pDNA was dissolved in Milli-Q water was stored at −20 °C prior to experiments. 

### 2.2. Preparation of Nanobubbles

Liposomes were prepared and the US contrast gas was encapsulated into the liposomes according to a previous study [13,15]. Briefly, distearoylphosphatidylcholine (DSPC) and Methoxypolyethyleneglycol 2000-distearoylphosphatidylethanolamine (PEG-DSPE) were dissolved in methanol, separately. Then the mixture of lipids (DSPC:PEG-DSPE = 94:6 (m/m)) was dried by evaporation and vacuum desiccated. the lipid film was dispersed in phosphate-buffered saline at 65 °C. The liposomes were sonicated for 3 min using a tip sonicator. After that, the liposomes were sterilized with a 0.45-mm filter. nanobubbles (2 mL of 1 mg/mL in 5 mL vial dispersion) were prepared from the liposomes. As the condition of the BLs production, the liposome enclosed with a 7.5 mL perfluoropropane gas was sonicated in a bath-type sonicator 1510 J-DTH (42 kHz, 90W, 5 min; Branson Ultrasonics, Tokyo, Japan). The particle size was measured using Zetasizer Nano ZS (Malvern Instruments, Worcestershire, UK). The z-average particle size was 314.3 ± 8.392 nm (*n* = 3). The particle size distribution and stability of nanobubbles was confirmed same as in our previous study [15].

### 2.3. Animals

Six-week-old male ddY mice (28–34 g) were purchased from Kiwa Laboratory Animals Co. Ltd. (Wakayama, Japan) and were housed in a cage in an air-conditioned room and maintained on a standard laboratory diet (CE-2, CLEA, Co., Ltd., Tokyo, Japan) and water. All animal experiments were carried out in accordance with the guidelines for animal experimentation of Nagasaki University (approval number: 1308051086-6 (2016)). 

### 2.4. Direct Sonoporation Method into the Peritoneum from the Peritoneal Tissue Surface

**Figure 1:**
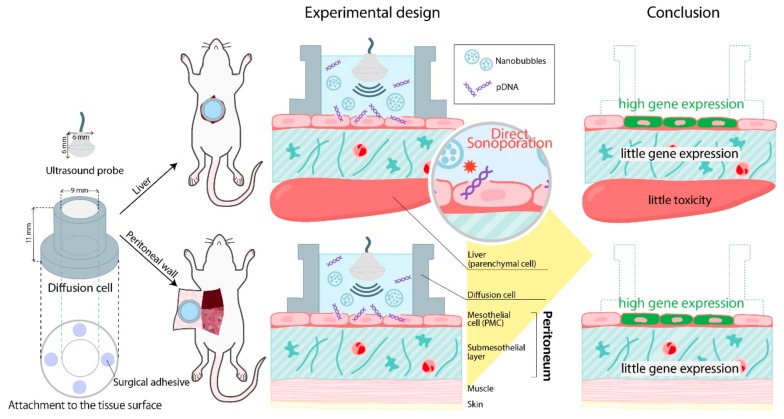
Scheme of the direct sonoporation to the defined area of the peritoneum from the peritoneal tissues. A glass-made cylindrical diffusion cell (in diameter: 9 mm) was attached to the peritoneal tissue surface by using surgical adhesive. A mixture consisting of pDNA and nanobubbles was directly added to the diffusion cell. Five min after administration, US (frequency, 2.04 MHz; duty, 50%; burst rate,10 Hz; intensity, 4.0 W/cm^2^) was irradiated using a Sonopore-4000 with a probe (diameter: 6 mm). Thus, direct sonoporation might be a site-specific transfection method into the peritoneal mesothelial cells (PMCs) on the applied peritoneum from the peritoneal tissue. In addition, under the PMCs, direct sonoporation showed little transgene expression and toxicity.

Figure 1 shows a schematic representation of the direct sonoporation system into peritoneum from the peritoneal tissue surface. Mice were anesthetized using an intraperitoneal injection that contained three types of mixed anesthetic agents (0.5 mg/kg of medetomidine, 4.0 mg/kg of midazolam, and 5.0 mg/kg of butorphanol) [28]. To apply it to the liver, the central abdomen was dissected by approximately 2 cm and a glass-made cylindrical diffusion cell (diameter: 9 mm, effective area: 63.6 mm^2^) was attached to the surface of the left lateral lobe of the liver with a surgical adhesive (Aron Alpha, Sankyo Co., Ltd., Tokyo, Japan). To apply it to the peritoneal wall, the central abdomen was dissected and turned over the peritoneal wall such that the parietal peritoneum was exposed. Then, the diffusion cell was attached to the surface of the left peritoneal wall. The mixture consisting of pDNA in saline and nanobubbles was directly added to the diffusion cell. At 5 min after administration, US (frequency, 2.04 MHz; duty, 50%; burst rate,10 Hz; intensity, 4.0 W/cm^2^) was irradiated into the mixture toward the liver surface using a Sonopore-4000 (Nepa Gene, Chiba, Japan) with a KP-L6 L-sharp 6 mm transducer (Nepa Gene, Chiba, Japan) according to a previous study [29]. After 10 min, the pDNA solution was removed from the diffusion cell, and the surface area of liver attached with the diffusion cell was washed with 500 µL of saline five times. Then, the diffusion cell was removed from the tissue surface and the abdomen was sutured. Mice were kept lying supine for 1 h and then freed in the cage.

### 2.5. Luciferase Assay

To determine transgene expression, the luciferase activity was measured as described previously [30]. Luciferase activity was indicated as relative light units (RLU) per g tissue. The average weight of applied site and non-applied site of liver was 350.9 ± 59.7 mg and 1078.5 ± 154.2 mg, respectively. The average weight of applied site and non-applied site of peritoneal wall was 194.3 ± 52.5 mg and 198.8 ± 37.7 mg, respectively.

### 2.6. Multicolor Deep Imaging Analysis

Multicolor deep imaging was performed as described previously [15]. Tissue clearing and stain the peritoneal surface cells were performed by Sca*l*eSQ (0) method [31] and intraperitoneal injection of 1,10-Dioctadecyl-3,3,30,30-tetramethylindocarbocyanine perchlorate (DiI) [15], respectively. Nuclei were stained by following as previously [30]. Clearing and staining tissue was observed by inverted confocal microscope (LSM 710 with spectral imaging equipment, Carl Zeiss Microimaging GmbH, Jena, Germany). The acquisition software was ZEN 2012. Objective lenses were a 20× dry lens (EC Plan-Neofluar, numerical aperture (NA): 0.5; working distance 17(WD): 2.0 mm) and 40× oil-immersion lens (EC Plan-Neofluar, NA: 1.30 WD: 0.21 mm).

To identification of transgene-positive cell, we conducted the 3 dimensional (3D)-immunohistochemistry by CUBIC method [32]. To observe the mesothelial cells, we used a 1:450 dilution of anti-mesothelin antibody (Santa Cruz Biotechnology, Inc., Santa Cruz, CA, USA) as primary antibody and a 1:750 dilution of Alexa Fluor® 647-conjugated goat anti-rabbit IgG antibody (Abcam, Cambridge, MA, USA) as secondly antibody.

### 2.7. Histological Evaluation

The liver tissues were stained with hematoxylin and eosin (H&E). The stained samples were observed as described previously [33]. In a hydrodynamics-based procedure as a positive control, a high volume of pDNA solution (1.6 mL per mouse) was injected into the tail vein within 5 s [34].

### 2.8. Assessment of Serum Alanine Aminotransferase (ALT) Level

To assess hepatic toxicity caused by direct sonoporation, serum alanine ALT activity was determined as described previously [15].

### 2.9. Statistical Analysis

Statistical comparisons were performed using the Student’s *t*-test for two groups and Tukey’s or Dunnett’s test for multiple groups. A value of *p* < 0.05 was considered significant.

## 3. Results

### 3.1. Effects of Direct Sonoporation to the Visceral Peritoneum from the Liver Surface

**Figure 2:**
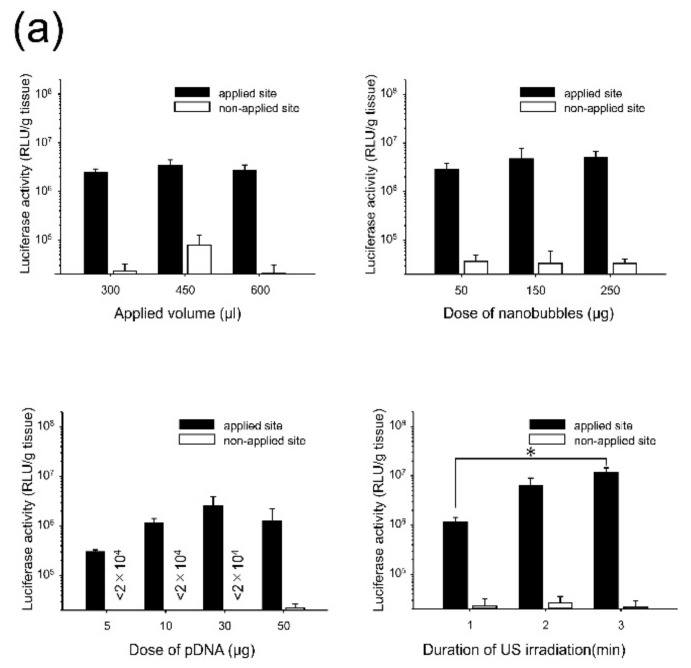

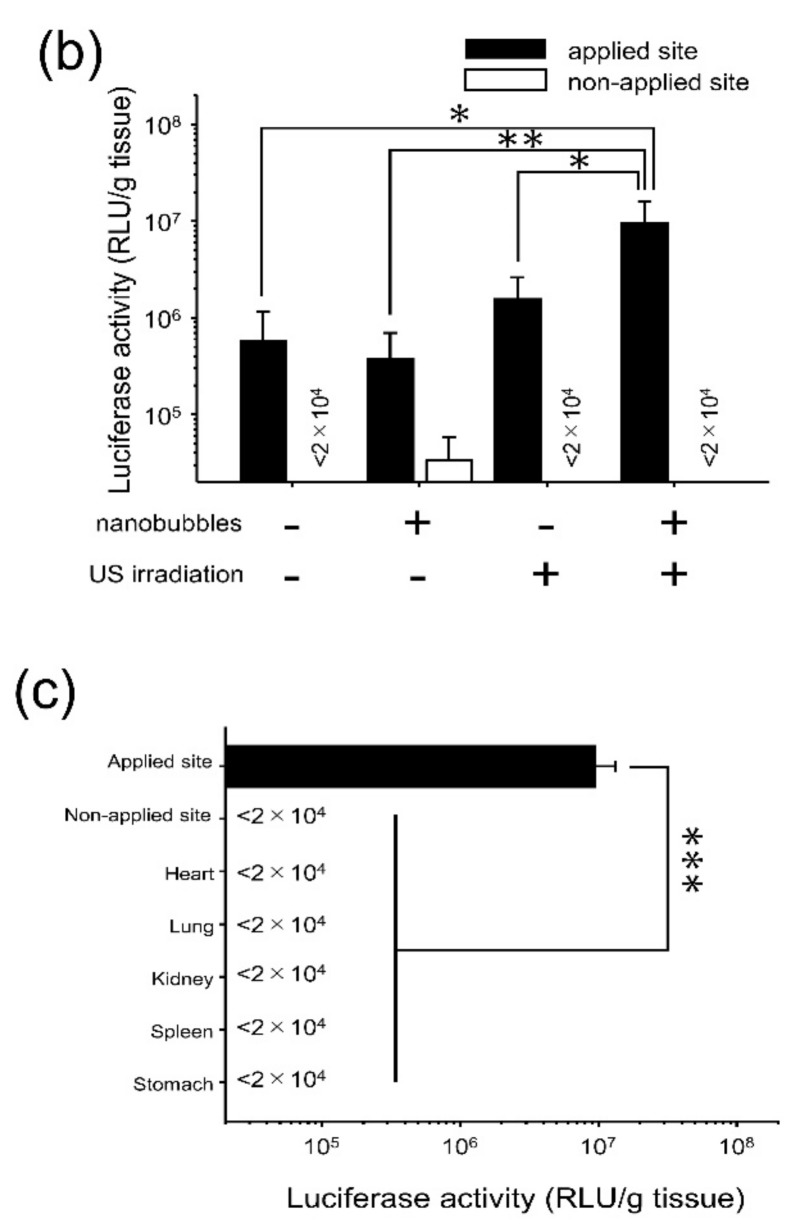
Effect of direct sonoporation to the visceral peritoneum from the liver surface. (**a**) Effects of the transfection conditions (applied volume, dose of nanobubbles, dose of pDNA, and duration of US irradiation). Administration of the mixture of CMV-Luciferase (the firefly luciferase gene expression vector driven by a cytomegalovirus (CMV) promoter) and nanobubbles followed by ultrasound (US) irradiation. Data is represented as mean + SE of 5–6 experiments. Statistical comparison was performed by Tukey’s multiple comparison test (* *p* < 0.05). (**b**–**c**): Transfection efficacy (**b**) and site-specificity (**c**) by direct sonoporation under the optimized conditions. Three-hundred microliters of the mixture of pCMV-Luciferase (30 µg) and nanobubbles (50 μg) was administrated to the applied site of the liver surface, then US were irradiated (4.0 W/cm^2^) for 3 min. Data is represented as mean + SD of 3–4 experiments. Statistical comparison was performed by Tukey’s multiple comparison test (**b**) and Dunnett’s multiple comparison test (**c**). (* *p* < 0.05, ** *p* < 0.01, *** *p* < 0.001)

To obtain effective gene transfection at the applied site of the liver, effects of the transgene conditions such as the applied volume, dose of the nanobubbles, pDNA, and duration of US irradiation were evaluated (Figure 2a). Because our previous studies have shown that the activity of nanobubbles under the US irradiation is disappeared until about 3 min [15,35], in this study, we evaluated effect of US duration time within 3 min. Transgene expression was not affected by the administration volume (300–600 µL) and the dose of the nanobubbles (50–250 µg). With respect to the dose of pDNA, transgene expression at the applied site increased up to 30 µg and remained constant thereafter. Moreover, transgene expression at the applied visceral peritoneum site increased depending on the duration of the US irradiation. The transgene expression for 3 min of US irradiation was significantly higher than that for 1 min of US irradiation. Then, the transfection efficacy and applied site-specificity in the visceral peritoneum by the direct sonoporation was also evaluated. Transgene expression at the applied site increased approximately by 100-fold using nanobubbles with US irradiation (Figure 2b). With respect to site-specificity, transgene expression at the applied site was significantly higher than the other sites and tissues (Figure 2c). 

### 3.2. Characterization of Transfection Expression by Direct Sonoporation to the Visceral Peritoneum from the Liver Surface

**Figure 3:**
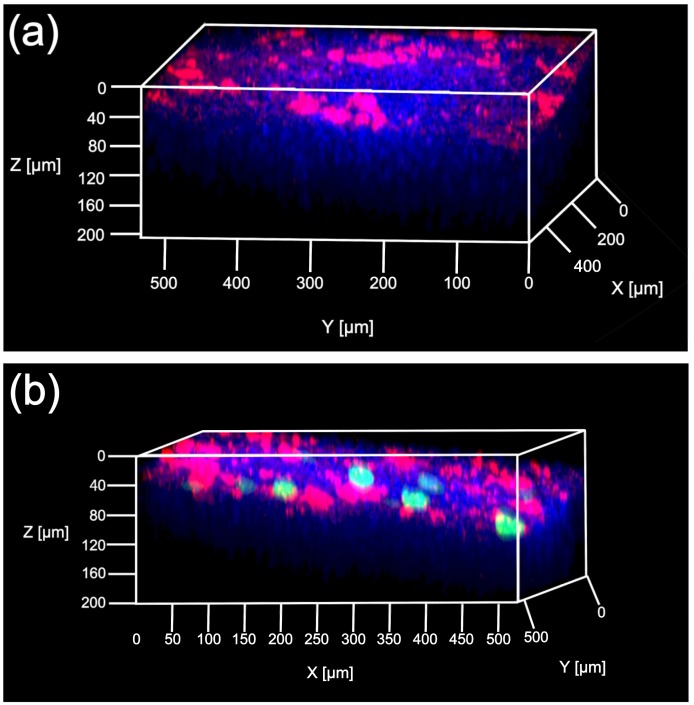
Evaluation of spatial distribution of transgene expression by direct sonoporation to the visceral peritoneum from the liver surface. Three-hundred microliters of a mixture containing pZsGreen1-N1 (30 µg) and nanobubbles (50 μg) was administrated to the applied site of the liver surface and US were irradiated (4.0 W/cm^2^) for 3 min. A view of the 3D observation at the non-applied site (**a**) and the applied site (**b**) after pZsGreen1-N1 transfection. Blue, green, and red signal show nuclei, transgene expression of pZsGreen1-N1 and DiI-stained surface cells. Objective lens used was 20x dry lens.

**Figure 4:**
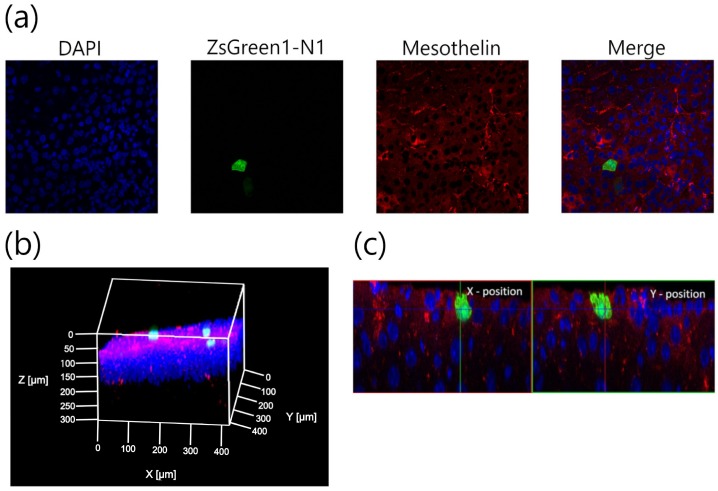
Identification of transgene-positive cell after pZsGreen1-N1 (green) transfection by direct sonoporation to the visceral peritoneum from the liver surface. Blue, green, and red signal show nuclei, transgene expression of pZsGreen1-N1 and Alexa-647^®^ (anti-mesothelin antibody). Observation was done using the 40× oil-immersion lens as 2D image (**a**), 3D image (**b**) and X-Z and Y-Z plane image (**c**).

Sonoporation can open the cell–cell junction of endothelial cell or blood–brain barrier by cavitation energy [26]. Therefore, it is possible that direct sonoporation open the cell-cell junction of mesothelial cells, then gene was transferred to the submesothelial layer. Therefore, we evaluate the depth of transgene expression from tissue surface to confirm whether transgene expression is over the PMCs layer. The spatial distribution of the transgene expression was evaluated using multi-color deep imaging analysis. Transgene expression was observed only at the applied site. Moreover, the transgene expression was defined in the surface cells of the liver stained by DiI (Figure 3). To understand the distribution of the transgene expression further, the transgene-positive cells were evaluated using 3D-immunohistochemistory (Figure 4). The anti-mesothelin antibody was used to stain the PMCs. Currently, transgene expression was observed at the region containing the PMCs layer by direct sonoporation to the visceral peritoneum from the liver surface.

### 3.3. Assessment of Hepatic Toxicity Caused by Direct Sonoporation to the Visceral Peritoneum from the Liver Surface

**Figure 5:**
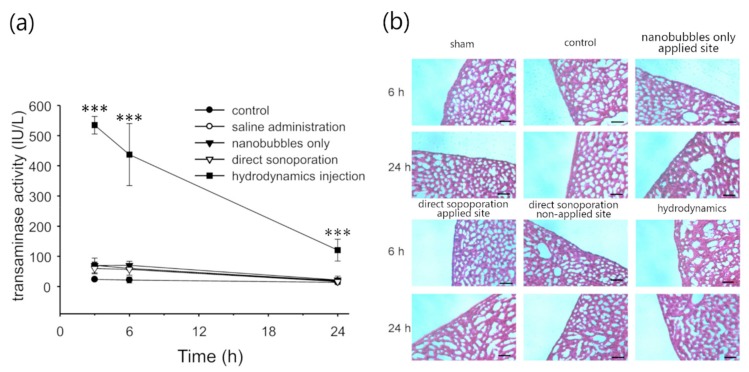
Assessment of liver impairments after direct sonoporation to the visceral peritoneum from the liver surface. (**a**) Serum alanine aminotransferase (ALT) activities after transfection. ALT activities were determined at 3, 6, and 24 h after each treatment. Each value is represented as mean ± SD of 4 experiments. *** *p* < 0.01 vs sham (open surgery) on Tukey’s multiple test. (**b**) H&E staining of the liver. These evaluations were performed at 6 h after treatment and at 24 h after transfection. Scale bar represents 100 µm.

To assess the toxicity caused by direct sonoporation to the visceral peritoneum from the liver surface, change in the histopathology of the applied site and the non-applied site was observed and evaluated using serum ALT activity and H&E staining. According to the serum ALT activity, the data obtained from the direct sonoporation group was not significantly different compared to the control group (Figure 5a). After 24 h of transfection by direct sonoporation, serum ALT activity was almost similar to the control. Furthermore, direct sonoporation did not change the morphological appearance in both the peritoneum and parenchymal cells in the liver compared to the sham (open surgery) group (Figure 5b). Thus, hepatic toxicity was not observed using both the toxic marker and histological assay. 

### 3.4. Characterization of Transfection Expression by Direct Sonoporation to The Parietal Peritoneum from the Peritoneal Wall Surface

**Figure 6:**
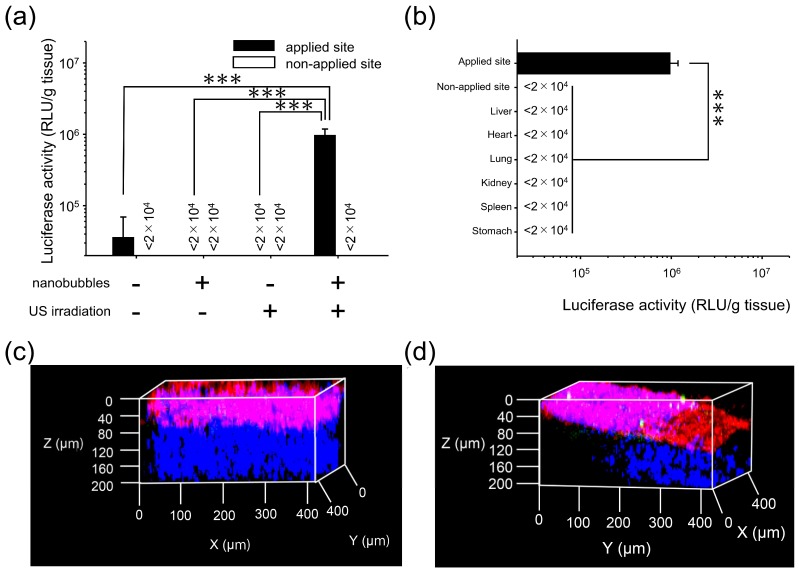
Characterization of the transgene expression by direct sonoporation of the parietal peritoneum from the peritoneal wall surface. a: transfection efficacy of the nanobubbles with US irradiation b: site-specificity. Thirty micrograms of pCMV-Luciferase and nanobubbles (50 µg) were administrated to applied site of the peritoneal wall surface, i.e., (**a**) total volume of 300 µL. Then, US was irradiated 4.0 W/cm^2^ for 3 min. Six hours after transfection, the luciferase activity was measured. Data is represented as mean + SD of four experiments. Statistical comparison was performed by Tukey’s multiple comparison test (**a**) and Dunnett’s multiple comparison test (**b**) (*** *p* < 0.001). (**c**,**d**) the distribution of transgene expression on the parietal peritoneum by 3D observation. The depth of the transgene expression at the non-applied site (**c**) and the applied site (**d**) after pZsGreen1-N1 transfection. Blue, green, and red signal show nuclei, transgene expression of ZsGreen1-N1 and DiI-stained surface cells. The objective lens used was the 20× dry lens.

To verify whether direct sonoporation can be used to transfect the parietal peritoneum, direct sonoporation was applied to the parietal peritoneum from the peritoneal wall surface. Transgene expression at the applied site increased by direct sonoporation, i.e., application to the visceral peritoneum from the liver surface (Figure 6a). Moreover, to assess site-specificity, the transgene efficiency at the applied site, the non-applied site of the peritoneal wall, and the other tissues (heart, lung, kidney, spleen, and stomach) was evaluated. Transgene expression at the applied site was significantly higher than that in both the non-applied site and the other tissues (Figure 6b). Except for the applied site, transgene expression level was much lower than the detection limit. Moreover, the spatial distribution of the transgene expression by the direct sonoporation to parietal peritoneum from the peritoneal wall surface was evaluated. As a result, the transgene expression was observed only at the applied site. Moreover, the transgene expression was defined based on the surface area on the peritoneal wall stained by DiI (Figure 6c,d).

## 4. Discussion

During sonoporation, the cavitation energy of sonoporation generated by nanobubbles with US irradiation is commonly affected by several conditions such as the administration volume, duration of US, and dose of nanobubbles [36,37]. To obtain stable transgene efficacy and site-specificity at the defined area in the intraperitoneal tissues, the effect of direct sonoporation was explained. As shown in Figure 2a, the transgene expression was not affected by the administration volume and dose of nanobubbles. On the contrary, transgene expression increased depending on the dose of pDNA i.e., from 5 to 30 µg and the duration of US irradiation from 1 to 3 min. The result of duration of the US irradiation is compatible with the results of our previous report regarding intraperitoneal injection in mice [15]. In the following experiments, a mixture of 300 µL of pDNA (30 µg) and nanobubbles (50 µg) was administered to mice, and the mice were subsequently irradiated with US for 3 min. Next, the transfection efficacy and site-specificity at the applied site of the liver under the optical conditions were assessed. Intraperitoneally injection based sonoporation by nano/microbubbles with transdermal US irradiation transfected to the entire intraperitoneal tissues [15,16]. Moreover, in case of pDNA and nano/microbubble complex such as cationic bubblelipoplex [29], remaining complex after sonoporation could be transferred to the blood circulation and transfected in the undesired site. In contrast, direct sonoporation is expected to be appropriate site-selective gene transfection method because sonoporation was conduct from a defined area and pDNA was removed after sonoporation. The transfection efficacy at the applied site increased by direct sonoporation to the visceral peritoneum from the liver surface while transgene expression at the non-applied site (Figure 2b) and transgene expressions at other sites in the tissues were not detected (Figure 2c). Measurement of luciferase activity may be slightly less sensitivity of quantitation than radioactivity. However, luciferase assay showed lower sample-to-sample variability and longer half-life than radioactivity [38]. On the other hand, in the previous study, we confirmed that Luciferase activity as transgene expression and radioactivity as pDNA distribution is corresponded [29]. Moreover, we checked measurement data of luciferase activity is within detection range. Thus, measurement of luciferase activity can be enough as evaluation of transgene efficacy. These results indicate direct sonoporation can achieve site-specific transgene expression at the applied site. Nanobubbles with US irradiation can affect the spatial distribution of transgene expression in the peritoneal tissues [15]. To clarify the distribution of transgene expression by direct sonoporation, which is studied little so far, the transgene expression was observed by multi-color deep imaging analysis. To observe the depth from the peritoneal tissue surface, surface cells were stained using an intraperitoneal injection of DiI. The transgene expression of direct sonoporation at the applied visceral peritoneum site of the liver was observed into stained with DiI (Figure 3). Our previous study has shown sonoporation toward the entire peritoneal tissues transfected into the DiI-stained peritoneal tissue surface. Therefore, transgene expression may be in the PMCs on the applied visceral peritoneum site. However, Data obtained using the DiI staining technique may not be enough to identify a transgene positive-cell. Therefore, in the present study, we challenged the 3D-immunohistochemistry observation by staining the PMCs. To identify transgene positive-cells, PMCs were stained with anti-mesothelin antibodies. As a result, transgene expression was observed only in the region stained by the anti-mesothelin antibodies (Figure 4). These observations provide evidence that direct sonoporation into the visceral peritoneum from the liver surface can transfect the PMCs at the applied visceral peritoneum site specifically without causing transgene expression at other sites of the visceral peritoneum and inner tissues of the peritoneum.

Our previous studies have reported that there is little damage at the peritoneal tissues transfected by the sonoporation [15]. However, it is unclear about the injury to the inner tissues via cavitation energy caused by direct sonoporation to the peritoneum. Therefore, we evaluated injury by H&E staining and ALT activity following direct sonoporation to the visceral peritoneum on the liver surface. In Figure 5a, ALT activity of the hydrodynamic injection was significantly higher than any other group at every sampling point as a positive control (Figure 5a). In contrast, ALT activity after direct sonoporation group were not significantly different from the control group. Moreover, we evaluated a morphological change by H&E staining (Figure 5b). In the PMCs, submesothelial layer, and hepatic intrinsic region, the morphological change was not observed after the direct sonoporation as well as normal state. We evaluated the morphological change of 3 difference mice, the abnormality change was not observed after direct sonoporation. These results may indicate that direct sonoporation to the visceral peritoneum from tissue surface can show little toxicity in the inner tissues.

Considering the clinical use of the medical device, examination of the appropriate tissue for direct sonoporation is needed. Because not only are the tissue intrinsic cells present under the peritoneum but also the structure of the PMCs layer may be different between the visceral and the parietal peritoneum [39]. Thus, transfection characteristics could be altered based on the applied tissue. For example, hepatic parenchymal cells reside under the visceral peritoneum from the liver surface while muscle fiber reside under the parietal peritoneum from the peritoneal wall. Therefore, in the present study, we evaluated the transfection characteristics by direct sonoporation to the parietal peritoneum from the peritoneal wall surface. As a result, transfection efficacy of direct sonoporation was significantly higher than that of the pDNA-solo group, nanobubbles-solo group, and US irradiation-solo group at the applied site without transfection to other intraperitoneal tissues (Figure 6a,b). The transgene expression in the parietal peritoneum from the peritoneal wall surface was approximately tenfold lower than that of the visceral peritoneum from the liver surface. In addition, multi-color deep imaging analysis showed that transgene expression was observed only on the peritoneal wall surface cells as the PMCs (Figure 6c,d). These results suggest that direct sonoporation to the parietal peritoneum from the peritoneal wall surface enable site-specific transfection to the PMCs on the applied parietal peritoneum site. On the contrary, the reason for the difference in the transfection efficacy between the parietal and visceral peritoneum is unclear. However, the difference in the stiffness of the tissues might have caused this phenomenon. Moreover, the transfection efficacy for fiber-based tissues (e.g. muscle, stomach) by physical stimuli were lower than the other tissues [40,41]. Although there is a difference in the transfection efficacy, these results suggest that direct sonoporation via the peritoneal tissue surface transfected sufficient gene to both the visceral and parietal peritoneum.

## 5. Conclusions

We evaluated the application of direct sonoporation to the peritoneum from the defined surface area on peritoneal tissues in mice. Direct sonoporation achieved applied site-specific transgene expression locally at the defined area of the peritoneum. In addition, the multi-color deep imaging analysis revealed that the transgene expression can be in the PMCs. Importantly, the transgene expression and toxicity in the inner part of the peritoneum tissue cannot be observed. On the contrary, direct sonoporation could be applicable to both the visceral and parietal peritoneum, although we found that there was a little difference with respect to the efficacy of transgene expression. Our findings are valuable for the development of intraperitoneal sonoporation for the treatment of peritoneal refractory diseases such as fibrosis.
